# Invasive *Haemophilus influenzae* Disease, Europe, 1996–2006

**DOI:** 10.3201/eid1603.090290

**Published:** 2010-03

**Authors:** Shamez Ladhani, Mary P.E. Slack, Paul T. Heath, Anne von Gottberg, Manosree Chandra, Mary E. Ramsay, European Union Invasive Bacterial Infection Surveillance participants

**Affiliations:** Health Protection Agency, London, UK (S. Ladhani, M.P.E. Slack, M. Chandra, M.E. Ramsay); National Institute for Communicable Diseases, Johannesburg, South Africa (A. von Gottberg); St. George’s University of London, London (P.T. Heath)

**Keywords:** Haemophilus influenzae, Hib, conjugate vaccines, serotype replacement, epidemiology, surveillance, outcome, bacteria, podcast, research

## Abstract

An international collaboration was established in 1996 to monitor the impact of routine *Haemophilus influenzae* type b (Hib) vaccination on invasive *H. influenzae* disease; 14 countries routinely serotype all clinical isolates. Of the 10,081 invasive *H. influenzae* infections reported during 1996–2006, 4,466 (44%, incidence 0.28 infections/100,000 population) were due to noncapsulated *H. influenzae* (ncHi); 2,836 (28%, 0.15/100,000), to Hib; and 690 (7%, 0.036/100,000), to non–b encapsulated *H. influenzae*. Invasive ncHi infections occurred in older persons more often than Hib (median age 58 years vs. 5 years, p<0.0001) and were associated with higher case-fatality ratios (12% vs. 4%, p<0.0001), particularly in infants (17% vs. 3%, p<0.0001). Among non-b encapsulated *H. influenzae*, types f (72%) and e (21%) were responsible for almost all cases; the overall case-fatality rate was 9%. Thus, the incidence of invasive non–type b *H. influenzae* is now higher than that of Hib and is associated with higher case fatality.

## Abstract 2

Incidence and case-fatality ratios are higher for non–type b than for type b infection.

*Haemophilus influenzae* is differentiated according to its capsular polysaccharide composition into 6 serotypes (a–f) and noncapsulated strains (*1*). Before routine vaccination, *H. influenzae* type b (Hib) caused >80% of invasive *H. influenzae* infections, primarily in healthy children <5 years of age (*2*). In contrast, non–type b *H. influenzae* usually causes opportunistic infections (*3*–*7*), particularly among elderly persons, who often have predisposing medical conditions such as chronic respiratory disease or immunosuppression (*6*–*11*).

The introduction of the Hib conjugate vaccine into national childhood immunization programs in the 1990s has resulted in a marked and sustained reduction in the incidence of invasive Hib disease in many countries (*2*). However, concern exists about the long-term effectiveness of the Hib immunization programs (*12*) and possible disease replacement by other *H. influenzae* strains (*13*). Because Hib conjugate vaccine reduces pharyngeal carriage (*14*), other *H. influenzae* strains theoretically could take its place and cause invasive disease (*13*,*15*). Elimination of Hib carriage also may reduce natural boosting of immunity, thereby resulting in lower Hib antibody and increased susceptibility to invasive Hib disease in the long term (*16*).

In 1996, a collaborative surveillance network was established in Europe to describe the impact of routine Hib vaccination on the epidemiology of invasive *H. influenzae* disease. By 2006, a total of 28 countries participated in surveillance, and 14 countries, comprising an annual denominator population of 150 million persons, routinely serotyped all invasive clinical *H. influenzae* isolates. We describe the epidemiology of invasive *H. influenzae* disease in countries with established national Hib immunization programs, devoting particular attention to invasive non–type b *H. influenzae* disease.

## Methods

A 3-year European Union–funded study (the BIOMED II Hib surveillance project) was initiated in 1996 to study the epidemiology of invasive *H. influenzae* after the introduction of the Hib conjugate vaccine into national infant immunization programs. In 1998, this program was renamed European Union Invasive Bacterial Infection Surveillance (EU-IBIS) and expanded to include more countries; by 2006, a total of 28 countries routinely reported cases to EU-IBIS (www.euibis.org). Participating countries reported cases of invasive *H. influenzae* disease to a central database, with basic demographic details, clinical syndrome, outcome, specimen site, and serotyping data on isolates. The UK Health Protection Agency *Haemophilus* Reference Unit (HRU) coordinated development of standardized laboratory protocols for growing, serotyping, and PCR genotyping *H. influenzae* and exchange of clinical isolates to ensure consistency of results. HRU also provided genotypic confirmation of serotypes for countries without established reference facilities and regularly distributed quality assurance strains to participating laboratories to ensure comparability of results. Annual reports were made available to participants at meetings and on the EU-IBIS website.

Invasive *H. influenzae* disease was defined as isolation of the organism from a normally sterile site. A case of meningitis was defined as *H. influenzae* cultured from cerebrospinal fluid or clinical and/or radiologic features of meningitis with blood culture positive for *H. influenzae*. Other clinical presentations, including epiglottitis, pneumonia, cellulitis, and osteomyelitis, were defined as isolation of *H. influenzae* from a normally sterile site (usually blood cultures but occasionally from another sterile site, e.g., joint fluid in septic arthritis or pleural fluid in empyema) in a person with clinical signs and symptoms consistent with that presentation. Bacteremia was defined as growth of *H. influenzae* from blood cultures only, with no distinctive clinical syndrome identified.

This study included only countries that routinely vaccinated children against Hib before 2000 and serotyped at least 50% of all clinical isolates. Detailed surveillance methods for all participating countries are available at www.euibis.org. Germany and Israel reported data for children only and were included in some of the analyses. Within the United Kingdom, data from England and Wales were collected separately from Scotland because the surveillance program in England and Wales is separate from that in Scotland. In Greece, during 1996–2002, surveillance was limited to a single prefecture, Attiki, and provided data only for persons <15 years of age; after 2002, national data were available for Greece. Italy initially relied solely on laboratory reporting with voluntary notification of confirmed cases of meningitis, but a more active laboratory-based surveillance system was established in 8 Italian regions in 1997–1998 (Campania, Liguria, Lombardia, Piemonte, Puglia, Toscana, Trento, and Veneto), 7 regions in 1999–2002 (Lombardia was no longer included), and nationally thereafter. All data were collected as part of enhanced national surveillance and rendered anonymous at the source.

Data were entered by using Microsoft Excel (Microsoft, Redmond, WA, USA), and statistical analysis was performed by using Stata 8.0 (www.stata.com). Total and age-grouped population estimates used as denominators for incidence calculations were obtained either from the national statistics website of the relevant country or from EU-IBIS participants (*17*). The denominator for disease incidence in infants <1 month, 1–5 months, and 6–11 months of age was estimated by dividing the number of infants (<1 year of age) by 12 and multiplying by the number of months in each age group, respectively. For non–type b encapsulated *H. influenzae* infections, we combined data on children 5–14 years of age with data on adults 15–64 years of age because serotype distribution, clinical presentation, and outcomes were similar for these 2 age groups. To estimate any increase in incidence of non–type b *H. influenzae* disease during the study period, an overdispersed Poisson regression model was fitted for the number of non–type b cases with covariates for year and country. To allow for changes in population and proportion of total *H. influenzae* isolates serotyped by country and year, these variables were included in the model as offsets. To control for differences in the collection of death data, we estimated case-fatality ratios (CFRs) by using the number of reported deaths as the numerator and all cases, including those for which outcome was not reported, as the denominator. We calculated age-adjusted odds ratios (ORs) for noncapsulated *H. influenzae* (ncHi) and Hib using logistic regression; we inclulded age and serotype (e.g., ncHi vs. Hib, *H. influenzae* type e [Hie] vs. *H. influenzae* type f [Hif]) as independent variables. Ages are given as medians and interquartile ranges (IQRs) and compared by using the Mann-Whitney U test. Proportions were compared by using the χ^2^ test or Fisher exact test; continuous variables were compared by using Student *t* tests.

## Results

During 1996–2006, a total of 14 countries reported 10,081 invasive *H. influenzae* cases, of which 2,836 (28.1%) were Hib, 690 (7.8%) were other capsulated *H. influenzae*, and 4,466 (44.3%) were ncHi. For 125 (1.2%) cases, the isolates were identified as non–type b, but complete serotyping was not performed. Capsular serotype was not available for 1,964 (19.5%) isolates, mainly because the isolate was not available for typing at the reference laboratory (e.g., where the isolate could not be recultured). The crude overall annual incidence rates for invasive Hib, ncHi, and non–type b encapsulated *H. influenzae* infections were 0.15, 0.28, and 0.036 cases per 100,000 population (Table 1). After adjusting for the proportion of isolates not serotyped in each country per year and population changes over time, we found a small but statistically significant increase in the incidence of non–type b *H. influenzae* disease (3.6% per year; 95% confidence interval [CI] 2.1%–5.2%).

**Table 1:** Incidence of invasive Hib and non–type b *Haemophilus influenzae*, by country and year of infection, Europe 1996–2006*

Country	Incidence (no. cases)											
	1996	1997	1998	1999	2000	2001	2002	2003	2004	2005	2006	Total
Austria	–	–	–	–	–	–	0	0.04 (3)	0.06 (5)	0.06 (5)	0.07 (6)	0.05 (19)
England, Wales	0.36 (183)	0.40 (205)	0.47 (244)	0.39 (202)	0.48 (248)	0.52 (274)	0.52 (273)	0.57 (302)	0.46 (245)	0.62 (333)	0.65 (350)	0.50 (2859)
Finland	0.29 (15)	0.29 (15)	0.57 (29)	0.35 (18)	0.64 (33)	0.83 (43)	0.23 (12)	0.54 (28)	0.40 (21)	0.73 (38)	0.52 (27)	0.49 (279)
Greece	–	–	–	–	–	–	–	0	0.01 (1)	0	0	0.00 (1)
Iceland	–	–	–	1.08 (3)	0	0	0	0	0	0	0	0.13 (3)
Ireland	0.14 (5)	0.30 (11)	0.17 (6)	0.21 (8)	0.08 (3)	0.23 (9)	0.18 (7)	0.20 (8)	0.35 (14)	0.29 (12)	0.52 (22)	0.25 (105)
Italy	0.05 (26)	0.08 (45)	0.04 (23)	0.03 (15)	0.02 (11)	0.02 (10)	0.01 (3)	0.02 (11)	0.01 (4)	0.02 (11)	0.02 (12)	0.03 (171)
Malta	–	–	–	0	0	0	0	0	0.26 (1)	0	0	0.03 (1)
The Netherlands	0.41 (64)	0.44 (68)	0.45 (70)	0.36 (56)	0.38 (61)	0.44 (71)	0.43 (67)	0.64 (103)	0.49 (80)	0.61 (99)	0.61 (99)	0.48 (838)
Norway	–	–	–	1.51 (67)	1.12 (50)	1.04 (47)	1.39 (63)	0.92 (42)	0.83 (38)	0.85 (39)	1.12 (52)	1.10 (398)
Portugal	–	–	–	0.05 (5)	0.07 (7)	0.15 (15)	0.12 (12)	0.09 (9)	0.08 (8)	0.10 (11)	0.16 (17)	0.10 (84)
Scotland	–	–	–	0	0.04 (2)	0.10 (5)	0.34 (17)	0.55 (28)	0.61 (31)	0.65 (33)	0.57 (29)	0.36 (145)
Slovenia	–	–	–	–	0.30 (6)	0.80 (16)	0.35 (7)	0.60 (12)	0.60 (12)	0.40 (8)	0.60 (12)	0.52 (73)
Total non–type b *H. influenzae* incidence	0.22 (293)	0.26 (344)	0.28 (372)	0.24 (374)	0.27 (421)	0.31 (490)	0.28 (461)	0.31 (546)	0.26 (460)	0.33 (589)	0.35 (626)	0.28 (4,976)
Hib incidence	–	–	–	–	0.12 (182)	0.14 (212)	0.23 (376)	0.21 (380)	0.15 (268)	0.13 (239)	0.09 (168)	0.15 (1,825)
Population, millions	132.5	132.8	132.9	153.9	156.3	156.9	165.6	177.4	178.5	179.8	180.6	

*Per 100,000 population. Data for Attiki, Greece (1996–2002); Germany; and Israel were not included because their surveillance was limited to pediatric cases. Hib, *H. influenza* type b.

After 2000, when all countries included in the study had implemented the Hib vaccine into their national immunization programs, 7,211 *H. influenzae* cases occurred, including 2,005 (27.8%) Hib and 3,172 (44.0%) ncHi cases. Patient sex did not differ for Hib and ncHi infections (931/1,948 [47.8%] Hib cases in female patients and 1,519/3,116 [48.7%] ncHi cases in male patients; χ^2^ = 0.44; p = 0.51). However, a higher proportion of women in the 25–44-year age group developed invasive ncHi infection: 117/165 (70.9%) for those 25–34 years of age and 93/166 (56.0%) for those 35–44 years of age, compared with 1,310/2,785 (47.0%) for the other age groups. Women 25–44 years of age who had invasive ncHi infection also were more likely than men in the same age group to have bacteremia (173/288 [60.1%] vs. 69/175 [39.4%]; χ^2^ = 18.6; p<0.001). For Hib, sex was not associated with clinical presentation for persons in any age group.

Children with Hib disease were much younger than those with ncHi (median 4.5 years [IQR 1.5–46.3 years] vs. 58.2 [IQR 6.8–76.4]) years, p<0.0001) (Figure). More than half of Hib cases, compared with fewer than one quarter of ncHi cases, occurred in children <5 years of age (χ^2^ = 438; p<0.0001). By contrast, only 13.7% of Hib cases occurred in persons >65 years of age, compared with 42.7% of ncHi (χ^2^ = 483; p<0.0001). The most common clinical diagnosis was bacteremia for both Hib and ncHi, but the median age of persons with Hib bacteremia was much lower than for that for those with ncHi bacteremia (Table 2). Hib meningitis occurred mainly in infants, whereas ncHi meningitis occurred in all age groups. Overall, ncHi was responsible for one third of all meningitis cases but accounted for 18.4% of meningitis cases in children <5 years of age and for 62.9% of persons >65 years of age. Hib was responsible for 84.1% of epiglottitis cases; only 6.6% were caused by ncHi. Hib pneumonia occurred mainly in adults and elderly persons; ncHi pneumonia occurred more often in children <5 years of age and in elderly persons.

**Figure:**
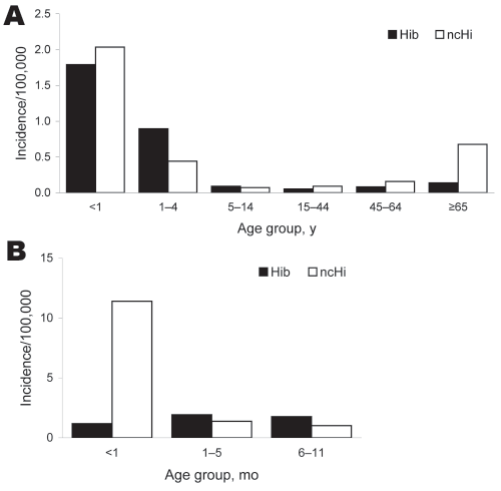
Age-specific incidence for disease caused by *Haemophilus influenzae* type b (Hib) and noncapsulated *H. influenzae* (ncHi) for all countries combined, Europe, 2000–2006. A) All age groups; B) infants <1 year of age.

**Table 2:** Hib and ncHi cases, by diagnosis and age group, Europe 1996–2006*

Diagnosis	Age group									NR	Total	Median age, y
	<1 mo	1–5 mo	6–11 mo	<1 y	1–4 y	5–14 y	15–44 y	45–64 y	>65 y			
Hib												
Meningitis	6 (31.6)	93 (60.4)	98 (57.6)	197 (57.4)	282 (41.2)	45 (23.0)	26 (10.0)	17 (7.1)	12 (4.4)	1 (12.5)	580 (28.9)	1.6
Epiglottitis	0	1 (0.6)	4 (2.4)	5 (1.5)	110 (16.1)	29 (14.8)	45 (17.4)	37 (15.4)	22 (8.0)	0	248 (12.4)	6.7
Cellulitis	0	7 (4.5)	18 (10.6)	25 (7.3)	15 (2.2)	3 (1.5)	0	1 (0.4)	4 (1.5)	0	48 (2.4)	1.0
OM/SA	0	4 (2.6)	5 (2.9)	9 (2.6)	24 (3.5)	4 (2.0)	4 (1.5)	6 (2.5)	1 (0.4)	0	48 (2.4)	2.1
Pneumonia	0	3 (1.9)	5 (2.9)	8 (2.3)	20 (2.9)	15 (7.7)	35 (13.5)	36 (14.9)	47 (17.2)	1 (12.5)	162 (8.1)	46.7
Bacteremia	11 (57.9)	33 (21.4)	30 (17.6)	74 (21.6)	193 (28.2)	73 (37.2)	102 (39.4)	96 (39.8)	134 (48.9)	2 (25.0)	674 (33.6)	13.6
Other	0	1 (0.6)	2 (1.2)	3 (0.9)	10 (1.5)	6 (3.1)	10 (3.9)	11 (4.6)	7 (2.6)	0	47 (2.3)	34.6
NR	2 (10.5)	12 (7.8)	8 (4.7)	22 (6.4)	30 (4.4)	21 (10.7)	37 (14.3)	37 (15.4)	47 (17.2)	4 (50.0)	198 (9.9)	39.0
All cases	19 (100.0)	154 (100.0)	170 (100.0)	343 (100.0)	684 (100.0)	196 (100.0)	259 (100.0)	241 (100.0)	274 (100.0)	8 (100.0)	2005 (100.0)	4.5
ncHi												
Meningitis	8 (4.4)	15 (13.6)	19 (19.4)	42 (10.8)	76 (22.6)	37 (24.2)	65 (14.3)	63 (13.6)	45 (3.3)	4 (20.0)	332 (10.5)	19.7
Epiglottitis	2 (1.1)	0	0	2 (0.5)	2 (0.6)	1 (0.7)	5 (1.1)	5 (1.1)	3 (0.2)	0	18 (0.6)	42.1
Cellulitis	0	0	1 (1.0)	1 (0.3)	4 (1.2)	5 (3.3)	0	0	4 (0.3)	0	14 (0.4)	11.6
OM/SA	0	0	0	0	2 (0.6)	1 (0.7)	2 (0.4)	2 (0.4)	4 (0.3)	0	11 (0.3)	56.6
Pneumonia	10 (5.5)	14 (12.7)	11 (11.2)	35 (9.0)	39 (11.6)	11 (7.2)	34 (7.5)	56 (12.1)	226 (16.7)	0	401 (12.6)	70.0
Bacteremia	129 (70.9)	53 (48.2)	46 (46.9)	228 (58.5)	151 (44.8)	74 (48.4)	236 (52.0)	218 (47.0)	759 (56.1)	10 (50.0)	1,676 (52.8)	59.6
Other	3 (1.6)	8 (7.3)	8 (8.2)	19 (4.9)	21 (6.2)	5 (3.3)	20 (4.4)	9 (1.9)	38 (2.8)	2 (10.0)	114 (3.6)	31.2
NR	30 (16.5)	20 (18.2)	13 (13.3)	63 (16.2)	42 (12.5)	19 (12.4)	92 (20.3)	111 (23.9)	275 (20.3)	4 (20.0)	606 (19.1)	61.3
All cases	182 (100.0)	110 (100.0)	98 (100.0)	390 (100.0)	337 (100.0)	153 (100.0)	454 (100.0)	464 (100.0)	1354 (100.0)	20 (100.0)	3,172 (100.0)	58.2

*Pediatric cases only for Attiki, Greece (1996–2002); Germany (1998 onward); and Israel (1996 onward). Values are no. (%) cases except as indicated. Hib, *Haemophilus influenzae* type b; ncHi, noncapsulated *H. influenzae*; NR, not recorded; OM/SA, osteomyelitis/septic arthritis.

In infants, the overall incidence of Hib and ncHi was similar, but incidence of the latter was much higher in the first month of life (11.4 vs. 1.2 cases per 100,000 population). In the first month, ncHi cases were more likely to occur in the first week (148/182 [81.3%]) compared with Hib (9/19 [47.4%] cases; χ^2^ = 11.6; p = 0.001); 112/148 (75.7%) of ncHi case-patients in the first week of life had bacteremia, compared with 50.0% (17/34) of those in whom ncHi occurred at 7–30 days of life (χ^2^ = 8.8; p = 0.003). Infections from ncHi in infants decreased after the first month of life and remained fairly constant during the first year (Figure).

A total of 585 (8.1%) of the 7,211 persons with *H. influenzae* died; CFRs were highest for persons >65 years of age and for infants. We found no association between death and sex or year of infection. In most age groups, CFRs were higher for ncHi than for Hib; the largest difference was for infants (Table 3). For both Hib and ncHi, the CFR was lower for meningitis than for other clinical presentations. The age-adjusted OR for death from ncHi compared with Hib was 2.4 (95% CI 1.9–3.1, p<0.0001) overall, 3.3 (95% CI 1.5–7.5; p = 0.004) for pneumonia, and 3.3 (95% CI 1.5–7.5; p = 0.004) for bacteremia. The OR for meningitis was not significant (OR 0.85, 95% CI 0.4–1.9 years; p = 0.68).

**Table 3:** Case-fatality rates for Hib and ncHi, by diagnosis and patient age group, Europe, 1996–2006*

Diagnosis	Age group									NR	Total
	<1 mo	1–5 mo	6–11 mo	<1 y	1–4 y	5–14 y	15–44 y	45–64 y	>65 y		
Hib											
Meningitis	1/6 (16.7)	3/93 (3.2)	2/98 (2.0)	6/197 (3.0)	11/282 (3.9)	4/45 (8.9)	0/26 (0)	1/17 (5.9)	2/12 (16.7)	0/1 (0)	24/580 (4.1)
Epiglottitis	–	0/1 (0)	0/4 (0)	0/5 (0)	3/110 (2.7)	1/29 (3.4)	2/45 (4.4)	2/37 (5.4)	0/22 (0)	–	8/248 (3.2)
Cellulitis	–	0/7 (0)	0/18 (0)	0/25 (0)	0/15 (0)	0/3 (0)	–	0/1 (0)	0/4 (0)	–	0/48 (0)
OM/SA	–	0/4 (0)	0/5 (0)	0/9 (0)	0/24 (0)	0/4 (0)	0/4 (0)	0/6 (0)	0/1 (0)	–	0/48 (0)
Pneumonia	–	0/3 (0)	0/5 (0)	0/8 (0)	0/20 (0)	0/15 (0)	1/35 (2.9)	1/36 (2.8)	5/47 (10.6)	1/1 (100)	8/162 (4.9)
Bacteremia	0/11 (0)	2/33 (6.1)	1/30 (3.3)	3/74 (4.1)	6/193 (3.1)	2/73 (2.7)	7/102 (6.9)	4/96 (4.2)	18/134 (13.4)	1/2 (50.0)	41/674 (6.1)
Other	–	1/1 (100.0)	0/2 (0)	1/3 (33.3)	1/10 (10.0)	0/6 (0)	2/10 (20.0)	0/11 (0)	0/7 (0)	–	4/47 (8.5)
NR	0/2 (0)	0/12 (0)	0/8 (0)	0/22 (0)	0/30 (0)	0/21 (0)	1/37 (2.7)	1/37 (2.7)	1/47 (2.1)	0/4 (0)	3/198 (1.5)
All cases	1/19 (5.3)	6/154 (3.9)	3/170 (1.8)	10/343 (2.9)	21/684 (3.1)	7/196 (3.6)	13/259 (5.0)	9/241 (3.7)	26/274 (9.5)	2/8 (25.0)	88/2,005 (4.4)
ncHi											
Meningitis	0/8 (0)	1/15 (6.7)	1/19 (5.3)	2/42 (4.8)	4/76 (5.3)	3/37 (8.1)	0/65 (0)	1/63 (1.6)	2/45 (4.4)	2/4 (50.0)	14/332 (4.2)
Epiglottitis	1/2 (50.0)	–	–	1/2 (50.0)	0/2 (0)	0/1 (0)	0/5 (0)	0/5 (0)	0/3 (0)	–	1/18 (5.6)
Cellulitis	–	–	0/1 (0)	0/1 (0)	0/4 (0)	0/5 (0)	–	–	1/4 (25.0)	–	1/14 (7.1)
OM/SA	–	–	–	–	0/2 (0)	0/1 (0)	0/2 (0)	0/2 (0)	0/4 (0)	–	0/11 (0)
Pneumonia	3/10 (30.0)	0/14 (0)	1/11 (9.1)	4/35 (11.4)	4/39 (10.3)	2/11 (18.2)	4/34 (11.8)	6/56 (10.7)	40/226 (17.7)	–	60/401 (15.0)
Bacteremia	22/129 (17.1)	18/53 (34.0)	0/46 (0)	40/228 (17.5)	14/151 (9.3)	2/74 (2.7)	11/236 (4.7)	22/218 (10.1)	131/759 (17.3)	2/10 (20.0)	222/1,676 (13.2)
Other	2/3 (66.7)	6/8 (75.0)	5/8 (62.5)	13/19 (68.4)	6/21 (28.6)	1/5 (20.0)	0/20 (0)	0/9 (0)	4/38 (10.5)	1/2 (50.0)	25/114 (21.9)
NR	1/30 (3.3)	5/20 (25.0)	2/13 (15.4)	8/63 (12.7)	3/42 (7.1)	0/19 (0)	1/92 (1.1)	7/111 (6.3)	24/275 (8.7)	0/4 (0)	43/606 (7.1)
All cases	29/182 (15.9)	30/110 (27.3)	9/98 (9.2)	68/390 (17.4)	31/337 (9.2)	8/153 (5.2)	16/454 (3.5)	36/464 (7.8)	202/1354 (14.9)	5/20 (25.0)	366/3,172 (11.5)

*Pediatric cases only for Attiki, Greece (1996–2002); Germany (1998 onward); and Israel (1996 onward). Values are no. deaths/no. cases (case-fatality rate). Hib, *Haemophilus influenzae* type b; ncHi, noncapsulated *H. influenzae*; OM/SA, osteomyelitis/septic arthritis; NR, not recorded.

Invasive infections caused by non–type b encapsulated *H. influenzae* were rare. Cases did not cluster by country or time, and individual serotypes or incidence, either overall or in any single country, did not increase during the 11-year study period. Of the 690 cases, Hif was the most prevalent subtype (500 [72.5%] patients) followed by Hie (143 [20.7%]) (Table 4). The overall CFR was 9.1% (63/690 patients). The CFR increased with age and was highest for bacteremia (36/292 [12.3%]) and pneumonia (15/127 [11.8%]) compared with meningitis (5/120 [4.2%]) and epiglottitis (0/10). CFR was highest for Hie infections (23/143 patients [16.1%]), particularly for persons >65 years of age (17/72 [23.6%]); however, none of the 22 children <16 years of age who had Hie infection died. Compared with Hif, the age-adjusted OR for death from Hie was 2.0 (95% CI 1.1–3.9; p = 0.035). All 3 Hia-related fatalities occurred in children <2 years who had meningitis.

**Table 4:** Epidemiology, diagnosis, and outcome of invasive non–type b *Haemophilus influenzae* infections, by serotype and age group, Europe, 1996–2006*

Data	Hia, n = 26	Hic, n = 9	Hid, n = 12	Hie, n = 143	Hif, n = 500	Total, N = 690
Diagnosis, no. (%) cases						
Bacteremia	5 (19.2)	1 (11.1)	0	44 (30.8)	157 (31.4)	207 (30.0)
Pneumonia	3 (11.5)	3 (33.3)	2 (16.7)	28 (19.6)	91 (18.2)	127 (18.4)
Meningitis	6 (23.1)	2 (22.2)	4 (33.3)	22 (15.4)	86 (17.2)	120 (17.4)
Other	6 (23.1)	0 (0)	1 (8.3)	30 (21.0)	87 (17.4)	124 (18.0)
Unknown	6 (23.1)	3 (33.3)	5 (41.7)	19 (13.3)	79 (15.8)	112 (16.2)
Total	26 (100.0)	9 (100.0)	12 (100.0)	143 (100.0)	500 (100.0)	690 (100.0)
Median age at disease onset, y (IQR)	2.1 (0.8–43.7)	52.0 (1.9–53.3)	36.5 (14.4–47.0)	65.3 (34.0–8.3)	61.0 (12.7–75.2)	60.7 (15.4–75.1)
Incidence/million cases (total no. cases) by age group, y						
<5	0.12 (17)	0.02 (3)	0	0.10 (14)	0.78 (106)	1.02 (140)
5–64	0.00 (4)	0.00 (5)	0.01 (10)	0.04 (57)	0.12 (171)	0.17 (247)
>65	0.02 (5)	0.00 (1)	0.01 (2)	0.25 (72)	0.77 (221)	1.04 (301)
All age groups	0.01 (26)	0.00 (9)	0.01 (12)	0.08 (143)	0.26 (500)†	0.36 (690)†
No. cases (%) by age group, y						
<5	17 (65.4)	3 (33.3)	0	14 (9.8)	106 (21.2)	140 (20.3)
5–64	4 (15.4)	5 (55.6)	10 (83.3)	57 (39.9)	171 (34.2)	247 (35.8)
>65	5 (19.2)	1 (11.1)	2 (16.7)	72 (50.3)	221 (44.2)	301 (43.6)
All age groups	26 (100.0)	9 (100.0)	12 (100.0)	143 (100.0)	500 (100.0)†	690 (100.0)†
No. (%) deaths by age group, y						
<5	3 (17.6)	0	–	0	3 (2.8)	6 (4.3)
5–64	0	1 (20.0)	0	6 (10.5)	11 (6.4)	18 (7.3)
>65	0	0	0	17 (23.6)	22 (10.0)	39 (13.0)
All age groups	3 (11.5)	1 (11.1)	0	23 (16.1)	36 (7.2)†	63 (9.1)†
Median age at death, y (IQR)	0.50 (0.47–2.3)	55.0‡	–	70.0 (63.8–88.9)	72.3 (55.9–83.4)	70.5 (51.7–83.7)

*Hia, *H. influenzae* type a; Hic, *H. influenzae* type c; Hid, *H. influenzae* type d; Hie, *H. influenzae* type e; Hif, *H. influenzae* type f; IQR, interquartile range. †Ages were not known for 2 persons with Hif infection. ‡Only 1 death was caused by this serotype.

The epidemiology of non–type b encapsulated *H. influenzae* varied with age. A total of 140 (20.4% of all infections) occurred in children <5 years of age. In this age group, meningitis (61 [43.6%] patients) was the most common clinical presentation and was caused by Hif (49 patients [80.3%]), Hie (7 patients [11.5%]), and Hia (5 patients [8.2%]). Children <5 years of age were more likely to have meningitis than were older children and adults (61/120 [50.8%] vs. 59/548 [10.8%]; χ^2^ = 83; p<0.0001). Two thirds of Hia infections (17/26 [65.4%] patients) occurred in children <5 years of age, compared with 21.2% (106/500) of Hif, 9.8% (14/143) of Hie, and 33.3% of Hic (3/9). Persons in this age group with either Hif (49/106 [46.2%] vs. 37/392 [9.4%]; χ^2^ = 79.0; p<0.0001) or Hie (7/14 [50.0%] vs. 15/129 [11.6%]; χ^2^ = 14.3; p<0.0001) infections were more likely to have meningitis, whereas older children and adults were more likely to have bacteremia and pneumonia. The CFR for children <5 years of age was lower than that for older children and adults (6/140 [4.3%] vs. 57/548 [10.4%]; χ^2^ = 5.0; p = 0.025).

Non–type b encapsulated *H. influenzae* infections among the 247 persons 5–64 years of age resulted mainly from Hif (17 [69.2%]) and Hie (57 [23.1%]). Most Hic (5/9 [55.6%]) and Hid (10/12 [83.3%]) infections also occurred in this age group. All serotypes were responsible for the 45 meningitis cases: Hif (26 [57.8%]), Hie (12 [26.7%]), Hid (4 [8.9%]), Hic (2 [4.4%]), and Hia (1 case [2.2%]). The CFR for this age group was 7.3% (18/247 patients), but no persons with meningitis died, compared with 12.0% (9/75 patients) and 11.5% (6/52 patients) of those with bacteremia and pneumonia, respectively.

Almost half the cases (301 [43.6%]) occurred among persons >65 years of age; Hif (221/301 [73.4%]) and Hie (72/301 [23.9%]) accounted for almost all cases. The overall CFR was highest for this age group (39/301 [13.0%] patients) and similar for those with bacteremia (17/102 [16.7%]), pneumonia (9/59 [15.3%]), or meningitis (2/14 [14.3%]). The CFR for Hie was 23.6% (17/72 patients), compared with 10.0% (22/221) for Hif (χ^2^ = 8.8; p = 0.003) for persons >65 years of age.

## Discussion

The marked reduction in invasive Hib disease after the introduction of the Hib conjugate vaccine had prompted concerns that other *H. influenzae* serotypes, ncHi, or other respiratory pathogens might fill the ecologic niche. However, little evidence exists for a substantial or sustained increase in invasive non–type b *H. influenzae* infections (*18*). The rise in Hib incidence during 2000–2002 resulted mainly from an increase in the United Kingdom and the Netherlands; however, rates remained well below those in the prevaccine era (*19**,**20*).

Although prospective enhanced national surveillance may be incomplete (*21*), comparisons over time and across serotypes are largely valid, assuming serotyping is accurate and complete. In addition, although lower ascertainment might lead to lower estimation of the true incidence of invasive *H. influenzae* disease, it is less likely to affect the clinical presentation, age distribution, outcome, or proportion of cases due to the different serotypes. All participating countries had reference laboratories that routinely serotyped all invasive *H. influenzae* strains and participated in an external quality assurance scheme. As a result, 80.5% of 10,081 *H. influenzae* isolates identified were serotyped. The robustness of the surveillance is demonstrated by the incidence of invasive non–type b disease, which remained relatively constant over the 11-year study period despite increasing numbers of participating countries and provides further confidence that replacement disease is not occurring (*18*,*22*,*23*).

In countries with established Hib immunization programs, the incidence of ncHi is now higher than that of Hib. Unlike Hib, however, invasive ncHi infections occur mainly in neonates and elderly persons. Neonatal ncHi infections are well described but account for <5% of all neonatal invasive bacterial infections (*3*,*24*,*25*). The infection develops rapidly (usually within 48 hours after birth) and follows a fulminant course with a high CFR, particularly in preterm infants (*3*). Invasive ncHi disease in neonates also is associated with septicemia in the mother, increased complications during labor, and preterm delivery (*24*,*26*,*27*). In our study, ncHi was more common among women in the 25–44-year age group, suggesting that childbearing-aged women may be at increased risk for invasive ncHi infections. This finding may reflect increased exposure, for example, because of contact with children or increased susceptibility, such as in pregnancy. In older children and adults who develop invasive ncHi infections, studies have reported that more than half the case-patients had serious predisposing medical conditions, such as chronic respiratory disease and impaired immunity (*3*–*7*). Unfortunately, because clinical information collected for individual cases in our study was limited, we could not further elucidate possible risk factors for invasive infections caused by the different serotypes.

Infection from non–type b encapsulated *H. influenzae* is extremely rare and mostly caused by Hif and Hie. Other population-based studies also have reported a predominance of Hif and, to a lesser extent, Hie among non–type b encapsulated *H. influenzae* in adults and children (*3*,*6*,*28*). The clinical presentations of both Hif and Hie disease are almost identical and similar to that of ncHi infections in that almost half the cases occurred among persons >65 years of age who usually had bacteremia and pneumonia (*6*,*7*). Although 44% of Hif infections occurred among persons >65 years of age, compared with 21% among children <5 years, the incidence was almost the same in the 2 age groups. Hif and Hie have considerably restricted genetic diversity, and most infections are caused by a few strains that may be intrinsically more pathogenic than noninvasive strains (*29*). Other studies have reported that, as with ncHi, 60%–80% of persons with invasive Hif disease and Hie had underlying conditions that predisposed them to opportunistic infections (*3**,**6**,**9**–**11**,**25**,**30**–**32*).

In contrast to Hif and Hie, invasive Hia infections were similar to Hib infection in that they occurred mainly in young children who often had meningitis (*2*). In our study, the incidence of Hia in children <5 years of age (0.12/million) was much lower than that reported in Navajo and White Mountain Apache children <5 years of age (20 cases/100,000 population) (*33*), Alaska Native children <2 years of age (21/100,000) (*34*), and northern Canadian aboriginal children <2 years of age (102/100,000) (*34*). The same populations are also highly susceptible to invasive Hib disease (*35*,*36*). Hia and Hib have the most closely related capsules (*37*) and a similar degree of genetic diversity (*29*).

Infections caused by Hic and Hid are rare and have low CFRs, suggesting that they may not be particularly virulent. There is a paucity of information on infections caused by these serotypes, even in the form of individual case reports. Our data suggest that these invasive Hic and Hid infections are more common in adults. A recent US study reported that, of 770 cases of invasive *H. influenzae* disease during 1996–2004 in Illinois, 3 (43%) of 7 Hic, and 4 (67%) of 6 Hid cases occurred in persons 18–64 years of age (*28*).

That CFR from invasive Hib disease remains low and similar to that reported in other industrialized countries is reassuring, even though it has not changed substantially from the prevaccine era (*2*,*38*). In contrast, CFRs for ncHi and non–type b encapsulated *H. influenzae* were significantly higher than for Hib. The CFRs in our study should be considered a minimum because we cannot be sure that all deaths would be reported to the surveillance systems, particularly if death occurred a considerable time after infection. Other studies with more active follow-up in adults with ncHi infections have reported CFRs of 13%–20% (*28*) and up to 29% within 1 month after infection (*7*). Although the high CFRs associated with early-onset neonatal ncHi is well described (*3*), our finding of such high CFRs in infants was unexpected. Whether these infants had any underlying medical conditions that predisposed them to death or the organisms causing infection in this age group are more virulent is not known. The CFR for invasive non–type b encapsulated *H. influenzae* infections was also higher than for Hib and comparable to the 15%–30% reported in other studies (*7*,*28*). The higher CFR for invasive ncHi infections among elderly persons and persons with other clinical diagnoses (Table 3) most likely results from a higher prevalence of underlying medical conditions predisposing them to opportunistic infections. In the latter group, for example, ncHi was often isolated from uncommon sterile sites, such as peritoneal and pericardial fluid, renal and spleen biopsy specimens, and brain abscesses, suggesting that such persons may have serious underlying medical conditions at the time of infection. Underlying medical conditions also may explain why CFRs may be higher for persons with invasive Hie infections; the small number of Hie cases compared with Hib, ncHi, or Hif suggests that this serotype is not particularly virulent. Other studies have reported higher CFRs for Hie than for Hif, particularly for elderly persons (*28*).

Thus, despite the reduction in Hib disease, continued surveillance is needed for all *H. influenzae* infections across all age groups to assess the long-term effectiveness of Hib vaccination, rapidly detect unexpected population effects and potential changes in circulating strains, and monitor changes in the epidemiology of invasive *H. influenzae* disease. Further studies are needed to define more clearly host and pathogen risk factors for invasive *H. influenzae* infection and factors associated with death.
